# Via mucosa incision EUS-guided sampling for the diagnosis of conventional endoscopic biopsy-negative gastric wall thickening

**DOI:** 10.1038/s41598-017-16080-3

**Published:** 2017-11-21

**Authors:** Hongbo Shan, Xiaoyan Gao, Guangyu Luo, Jieqing Xiang, Bilv Zhong, Xiaofang Qiu, Shiyong Lin, Shuhong Li, Yin Li, Guoliang Xu, Rong Zhang

**Affiliations:** 1 Department of Endoscopy, Sun Yat-sen University Cancer Center, Guangzhou, 510060 China; 2 State Key Laboratory of Oncology in South China and Collaborative Innovation Center for Cancer Medicine, Sun Yat-sen University Cancer Center, Guangzhou, 510060 China

## Abstract

Abnormal thickened lesions of the gastric wall are usually covered with normal mucosa. Conventional endoscopic biopsies often do not yield sufficient positive histological results for clinical treatment. To increase the rate of diagnosis of conventional endoscopic biopsy-negative gastric wall thickening, we used an endoscopic submucosal dissection (ESD)-like sampling method under endoscopic ultrasound (EUS) guidance to obtain tissue of gastric wall-thickening lesions. Between 2012 and 2016, patients with gastric wall thickening (as identified by computed tomography (CT), EUS or other imaging methods that showed no positive findings in repeating conventional endoscopic biopsy) underwent via mucosa incision EUS-guided sampling. Final diagnosis was determined after surgical or biopsy pathology. A total of 10 patients with gastric wall thickening were included in this study. Eight cases received definite results, whereas in two cases the biopsy results were ambiguous and in these two patients poorly differentiated adenocarcinoma was determined by postoperative pathology. The results of the cases presented in this study demonstrated that via mucosa incision EUS-guided sampling provided a complementary option for the diagnosis of conventional endoscopic biopsy-negative gastric wall thickening.

## Introduction

Abnormal thickening of the gastric wall with or without gastric stenosis is often identified by a computed tomography (CT) scan or endoscopic ultrasound (EUS). The most common cause is subepithelial infiltrating malignance, such as linitis plastica^1^. Gastric wall thickening can also be caused by benign lesions, including eosinophilic infiltration or Ménétrier’s disease. Localized gastric wall thickening has also been observed in stromal tumors growing outward or in ectopic pancreas. Therefore, it is of utmost importance to sample the lesions for pathological examination to complete a definitive diagnosis.

Because the thickened lesions are usually covered with normal mucosa, a standard endoscopic biopsy often yields negative results. Bite-on-bite biopsy, an endoscopic technique commonly used in clinical settings, is not highly targeted and is associated with uncertain outcomes and a potential risk of perforation^2^. Unfortunately, it is not rare that patients with malignancies, especially linitis plastica, cannot be preoperatively diagnosed by multiple endoscopic biopsies. In the digestive tract, EUS-guided fine needle aspiration (EUS-FNA) is often used for cytological examination of lesions beneath the mucosa. However, inadequate sampling also causes negative or vague results^3^. Endoscopic submucosa dissection (ESD) is another approach for taking biopsies of the submucosal tumor. However, submucosal lesions are not clearly visible in the endoscopic view, which increases the risk of uncertain outcomes and potential gastric wall perforation^4,5^. In our clinical practice, we use via mucosa incision EUS-guided sampling as an ESD-like sampling means under EUS guidance. The advantage of this sampling approach is that once the submucosal lesion is located by convex EUS, a small mucosa incision is made similar as in the ESD-like technique, then forceps are inserted through the mucosal incision and a submucosal tunnel is created to sample the target lesion under EUS guidance. Adequacy of the tissue sample is guaranteed and the risk of perforation or injury of large submucosal vessels is prevented.

## Results

### Patient characteristics

A total of 10 patients who visited our hospital between 2012 and 2016 were enrolled in this study. Patients included 3 females and 7 males with an average age of 51.4 ± 15.2 years (range: 33–77 years) (Table 1).

**Table 1:** Characteristics of patients.

		N = 10
Male/female		7/3
Age, median (range), years		
40 (33–77)		
Characteristics of chief complaint		
	Dyspepsia	8
	Abdominal pain	5
	Abdominal distention	8
	Melena	3
	Abnormal finding of endoscopy or CT without noticeable symptoms	2
The first discovery method		
	CT	4
	Endoscopy/EUS	6
Locations of lesions		
	Gastric Corpus	3
	Gastric antrum	4
	Both	3

### Pathological results

A total of 5 out of 10 patients were found to have adenocarcinomas, of which 4 underwent surgery and 1 patient received chemotherapy. Moreover, 1 patient was found to have an ambiguous result and 1 patient yielded a negative result, however postoperative pathology results indicated that adenocarcinoma were present. In 2 patients, an ectopic pancreas was found with no signs of malignancies after half a year of surveillance. One patient was found with lipoma, which was proved by pathology results after surgery (Table 2).

**Table 2:** Diagnostic yields by via mucosa incision EUS-guided sampling.

Yields of sampling	Clinical management	Surgical pathology	N = 10
**Malignancy**			
Adenocarcinoma	Surgery	Adenocarcinoma	4
	Chemotherapy	—	1
**Ambiguous result**			
Suspicious tumor cells	Surgery	Adenocarcinoma	1
**Negative result**			
Granulation tissue	Surgery	Adenocarcinoma	1
**Benign tumor or disease**			
Lipoma	Surgery	Lipoma	1
Ectopic pancreas	Surveillances	—	2

All of cases showed negative results after multiple bite-on-bite mucosal biopsies.

### Post-procedure discomfort and complications

A total of 2 patients complained about abdominal pain, which significantly worsened after EUS-guided sampling compared to the pain before the procedure. The patients showed no positive signs of perforation during physical examination, and abdominal pain was significantly alleviated within 2 h after the procedure of EUS-guided sampling (Table 3). All patients who underwent biopsy experienced no massive gastric hemorrhage or perforation within the 5-day observation period.

**Table 3:** Post-procedure discomfort and complications.

	N = 10
Abdominal pain*	2/10
Massive hemorrhage of gastrointestinal tract	0/10
Perforation	0/10

^*^After via mucosa incision EUS-guided sampling, patients complained abdominal pain that significantly worsened pain compared to pain before procedure.

## Discussion

Abnormal thickening of the gastric wall is often identified during a CT scan and/or endoscopy/EUS examination. Some patients may present with several clinical symptoms, including difficulty swallowing, abdominal pain, bloating, and indigestion. However, other patients may present with no clear symptoms^1,6^. In some cases, gastric wall thickening can only be observed during CT scans when the gastric lumen is not well distended. In this study, several patients underwent biopsy because of additional concerns that were detected during CT imaging or EUS examination, such as wall stiffness, limitation of dilatancy, swelling of gastric folds, or destruction of gastric layers.

Adequate samples for diagnosis are of critical importance for clinical treatment, especially in malignancy. Our results showed that infiltrating malignant tumors, such as linitis plastic, are commonly found in gastric wall thickening. In most cases, bite-on-bite mucosal biopsy is a common approach to obtain a sufficient amount of tissue to make a definite diagnosis. However, some subepithelial lesions are deep-seated and may not be diagnosed despite multiple mucosal biopsies. In a study of by Jeong-Seoun Ji *et al*., the diagnosis of digestive tract wall thickening was identified in 54% of submucosal esophageal lesions. In case of gastric and duodenal lesions, diagnosis was lower, roughly 28%^2^. In our study, patients underwent via mucosa incision EUS-guided sampling of gastric tissue because of negative or ambiguous results after multiple bite-on-bite mucosal biopsies.

In the digestive tract, EUS-FNA can be used for cytological examination of lesions beneath the mucosa^3,7,8^. In a previous study, it was indicated that pro-core biopsy may be superior to fine needle aspiration^9^. However, in cases of gastric wall-thickening, the sampling inadequacy hinders the EUS-FNA approach. Kobara *et al*. reported that a gastric heterotopic pancreas could be identified by direct endoscopic imaging using submucosal endoscopy^10^. Chiyo *et al*. reported that submucosal endoscopic sampling was used for the diagnosis of gastric linitis plastic infiltrating into the submucosal layer^11^. Moreover, Binmoeller *et al*. introduced the Suck-ligate-unroof-biopsy (SLUB) technique for biopsy of subepithelial tumors^12^. When lesions show no obvious submucosal uplift lesions, the ESD-like biopsy may miss the lesion in the submucosal layer, leading to unexpected damage to the gastric wall.

**Figure 1:**
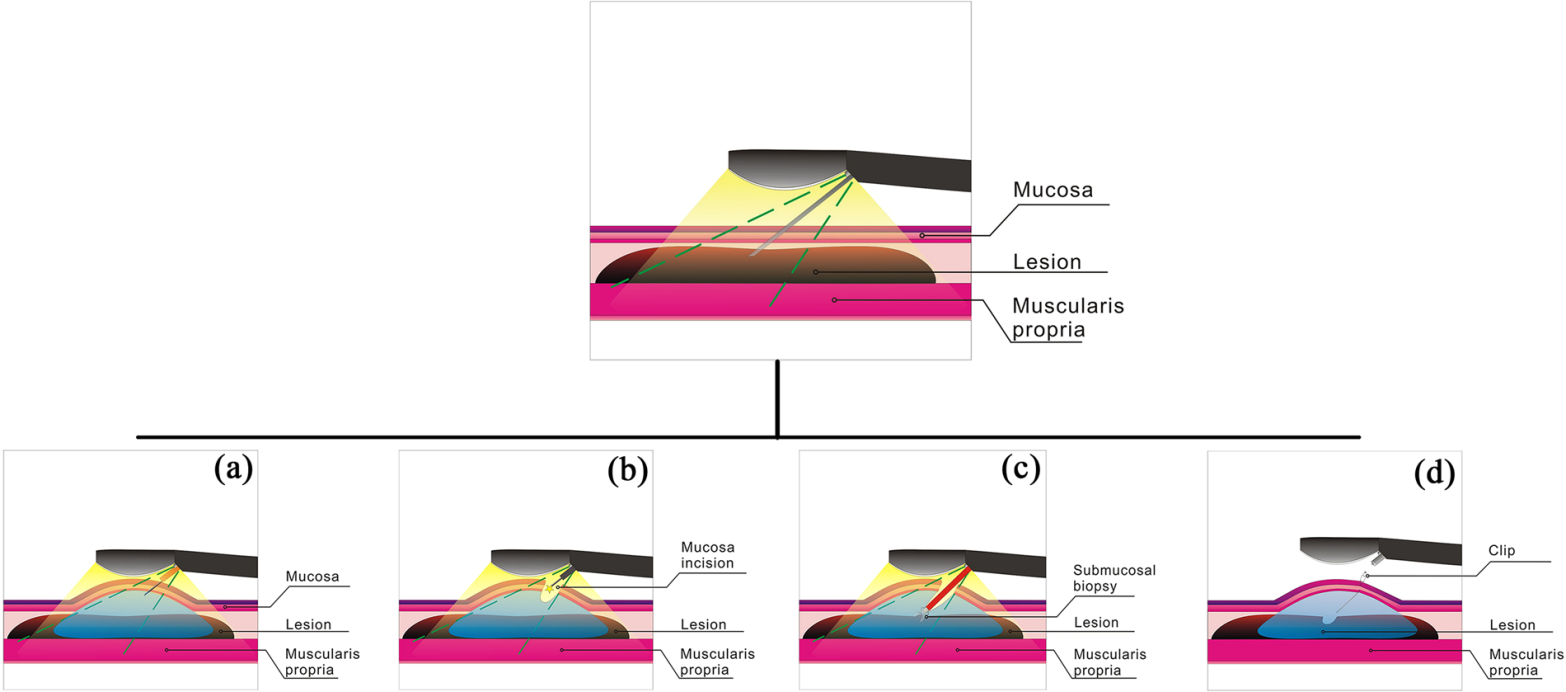
Illustration of the via mucosa incision EUS-guided submucosal sampling procedure. (**a**) Submucosal thickening of the gastric wall was identified using EUS guidance and normal saline was injected into submucosal tissue to prepare for mucosal incision; (**b**) The mucosal layer was dissected at an appropriate depth based on EUS evaluation; (**c**) The forceps are inserted though the incision using EUS monitoring and to biopsy the submucosal lesion; (**d**) The incision is closed with clips after biopsy.

In most cases, thickened walls vary in thickness, the thickest site being where the diseased tissue is concentrated. Mucosa incision EUS-guided sampling allows for taking samples in areas where the diseased tissue is most concentrated, which ensures sampling adequacy. In addition, unlike direct endoscopic imaging with submucosal endoscopy, mucosa incision EUS-guided sampling only requires a minimal mucosal incision of ~5 mm long in which the forceps are inserted. This is relatively safe and reliable under real-time EUS observation. The mucosa incision EUS-guided forceps biopsies could obtain adequate samples (see Fig. 1). In this study, mucosa incision EUS-guided sampling provided an alternative means for creating a more definitive pathological diagnosis of patients with gastric wall thickening, which significantly reduced uncertainty and therefore proved a valuable tool for clinical treatment. In this study, 1 out of 7 malignancy cases demonstrated a negative result of biopsy after EUS-guided sampling, which might be due to the stenosis of gastric antrum, the thickest site of lesion could not be reached.

Our data showed that via mucosa incision EUS-guided sampling is a feasible technique for obtaining gastric submucosal tissue and is considered safe and reliable with low complication rates. Via mucosa incision EUS-guided forceps biopsies benefits from the expertise of endoscopists who are familiar with both EUS-FNA skills and ESD skills. The post-procedure pain may relate to gastroenteric gas caused by endoscope introduction for which we used air for insufflation, and could be completely alleviated within 2 h after the EUS-guidance sampling procedure. In future studies, CO_2_ can be used for insufflation, which may have a potential impact on post-procedure pain. None of the patients experienced massive gastric hemorrhage or perforation within the 5-day observation period.

Therefore, our study established a potential method and strategy to diagnose the cases of conventional endoscopic biopsy-negative gastric wall thickening. However, this primary study was established in a single cancer center unit, and the results were limited to the target population who suffered from gastric wall thickening but presented with negative endoscopic biopsy results. The diagnostic efficiency of mucosa incision EUS-guided sampling for the diagnosis of biopsy-negative gastric wall thickening is promising but needs to be confirmed a larger scale.

In conclusion, for endoscopic biopsy-negative gastric wall thickening, via mucosa incision EUS-guided sampling can successfully be used to help obtain more samples. Therefore, it provides a powerful complementary means for making a definitive pathological diagnosis of patients with gastric wall thickening, especially for patients with suspicious malignancies.

## Methods

### Patient selection

#### Inclusion criteria

Patients who met the following criteria were included in this study: (1) patients who visited the Cancer Center of Sun Yat-sen University (Guangzhou, China) between 2012 and 2016, (2) patients who had gastric wall thickening as identified by CT, EUS, or other imaging methods and were suspected to have tumor lesions, especially malignancies, (3) patients who showed no positive findings in more than one conventional endoscopic biopsy, and (4) patients were classified as class 1 or 2 using the American Society of Anesthesiologists (ASA) physical status classification system.

#### Exclusion criteria

Patients who met any of the following criteria were excluded from this study: (1) patients who had severe cardiopulmonary dysfunction or serious disease of the liver or other vital organs, (2) patients who refused to be examined, (3) patients who underwent radiotherapy, chemotherapy or therapeutic surgery before examination. The study was approved by the Ethical Committee of Sun Yat-sen University Cancer Center, and written informed consent was provided by the patients (RDDA2017000271). All experiments were performed in accordance with relevant guidelines and regulations.

### Procedure

#### Preoperative preparation and sedation

Patients were fasted at least 6 h prior to the operation. A total of 10 mg of raceanisodamine was injected intramuscularly 10–15 min before the procedure, and 10 mg of diazepam was given to induce sedation. A total of 10 ml of dyclonine was administered orally to induce pharyngeal anesthesia.

#### Via mucosa incision EUS-guided sampling

Longitudinal convex EUS (EG-530UT, Fujinon, Tokyo, Japan or GF-UCT260, Olympus, Tokyo, Japan) was performed to facilitate access to the site of wall thickening. The mucosal incision was performed by using an ERBE 200D electrosurgical system (ERBE Elektro-medizin GmbH, Tubingen, Germany) and an Olympus Dual knife (Olympus, Tokyo, Japan). Firstly, submucosal saline injection was performed following a standard ESD-like procedure through the EUS channel. After a about 5-mm mucosal opening cut was made, a shallow submucosal tunnel was created. Then, submucosal tissue sampling was performed by using forceps through the submucosal tunnel at an appropriate depth under the EUS guidance. The incision was closed by clips after submucosal tissue sampling.

**Figure 2:**
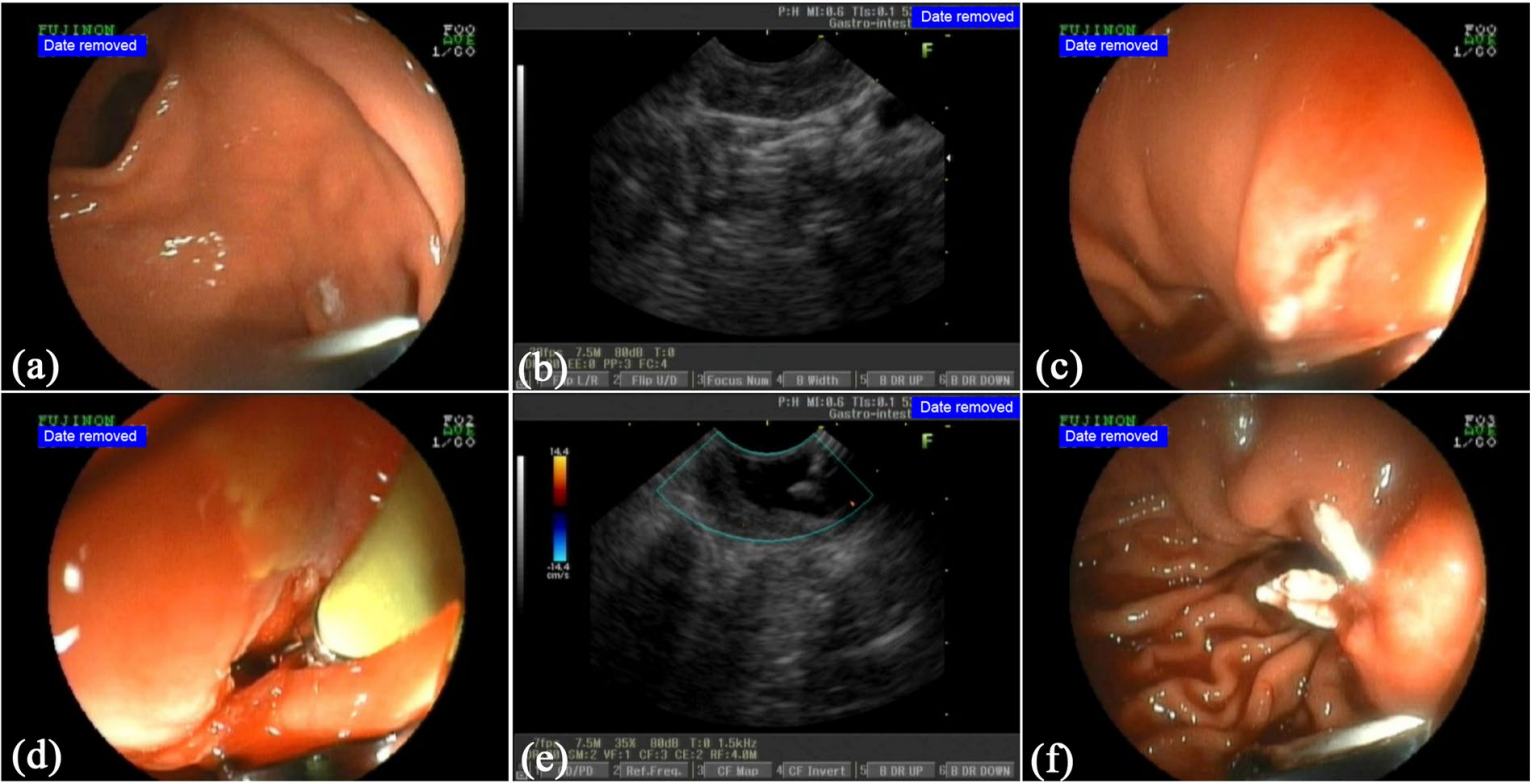
Case demonstration of the via mucosa incision EUS-guided sampling. A patient was found to have gastric wall thickening at the gastric antrum by CT. The mucosa biopsy yielded a negative result. The mucosa incision EUS-guided forceps biopsies made the diagnosis, ectopic pancreas; (**a**) An endoscopic view of the gastric antrum showed that the surface of the mucosa was smooth and had no obvious gastric submucosal uplift lesions; (**b**) An endoscopic sonography presented that the gastric wall thickening and the hyperechoic layer of submucosa was ambiguous. There was an isoechoic layer without a clear margin; (**c**) The mucosal layer was dissected at an appropriate depth based on EUS evaluation after submucosal administration with saline; (**d**) The forceps were inserted though the incision using monitoring of the endoscopic view; (**e**) To biopsy the submucosal lesion, the tip of the forceps were monitored by EUS; (**f**) After biopsy, the incision was closed with clips.

Figures 1(a to d) illustrate the procedure of EUS-guided mucosa incision and submucosal biopsy. Figure 2(a to f) demonstrate a case of the EUS-guided mucosa incision and submucosal biopsy.

#### Post-procedure management

After performing the via mucosa incision EUS-guided sampling, patients were hospitalized for 2 h for observation purposes. At any sign of massive gastric hemorrhage or perforation, the patient would be transferred to the hospital for emergency treatment. Patients received remote follow-up to observe any signs of tardive massive gastric hemorrhage or perforation. The total observation period was 5 days.

#### Pathological examination

Gastric tissue samples were embedded in paraffin, sectioned in 4um, and stained with hematoxylin and eosin (HE). All biopsy specimens were evaluated by pathologists of the Sun Yat-sen University Cancer Center (Guangzhou, China).

The stained sections were evaluated for (1) positivity (pathological diagnosis), (2) ambiguous (atypical and suspicious tumor cells), and (3) negativity (normal tissue or cells, such as chronic inflammatory cells).

#### Surgical pathology or surveillance

In cases of resectable malignancy tumor, patients underwent surgery and a postoperative pathological report was prepared to verify the diagnosis. Ambiguous or negative cases that were highly suspicious of malignancies also underwent surgical resection to obtain a pathology report. Cases in which resection of the tumor was not an option, received chemotherapy. In benign cases, such as mass-like ectopic pancreas or lipoma, after full informed consent, patients could choose surveillance by using endoscopy/EUS for half a year to prevent potential malignancy of the disease or complete lesion resection.
